# Draft Genome Sequence of Multidrug-Resistant Escherichia coli NIVEDI-P44, Isolated from a Chicken Fecal Sample in Northeast India

**DOI:** 10.1128/genomeA.00205-18

**Published:** 2018-04-26

**Authors:** Rituparna Tewari, Susweta Das Mitra, Sangita Das, Sudhir Jadhao, Gayatri Mishra, Feroze Ganaie, Rajeswari Shome, Habibur Rahman, Bibek Ranjan Shome

**Affiliations:** a ICAR-National Institute of Veterinary Epidemiology and Disease Informatics, Bengaluru, Karnataka, India; b Department of Microbiology, Jain University, Bengaluru, Karnataka, India; c School of Basic and Applied Sciences, Dayananda Sagar University, Bengaluru, Karnataka, India; d Bionivid Pvt Ltd., Bengaluru, Karnataka, India

## Abstract

We report here the draft genome sequence of a multidrug-resistant Escherichia coli strain (NIVEDI-P44) isolated from a chicken fecal sample. The estimated genome size is 4.76 Mb, with a G+C content of 50.65%. The genome harbors multiple antibiotic resistance genes, *bla*_DHA-1_, *mph*(A), *strA*, *strB*, *dfrA14*, *sul-2*, *tet*(A), and *qnrS1*.

## GENOME ANNOUNCEMENT

There is worldwide concern about the emergence and rapid rise of antibiotic-resistant Escherichia coli strains in human and veterinary medicine in both developed and developing countries (1). Although the gut of livestock, especially poultry, is an important reservoir for drug-resistant E. coli, these bacterial pathogens can be transmitted to humans through direct contact, food of animal origin, and environmental routes (2, 3). Whole-genome sequencing is considered essential in the epidemiological surveillance of multidrug-resistant (MDR) strains circulating in different hosts to decipher their resistome and transmission dynamics and to gain insights into their phylogenetic and phylodynamic aspects (4). Here, we report the draft genome sequence of the MDR E. coli strain NIVEDI-P44, recovered from a chicken fecal sample during our molecular surveillance study of extended-spectrum-β-lactamase- and carbapenemase-producing Gram-negative bacteria in farm animals from Northeast India. An MIC method utilizing a broth microdilution procedure revealed NIVEDI-P44 to be resistant to 7 different antibiotics (*viz*., ampicillin, tetracycline, ciprofloxacin, cefotaxime, cefotetan, ceftazidime, and ceftriaxone). These antibiotics are of human clinical relevance and are classified as “veterinary critically important antimicrobial agents” (VCIA) in veterinary medicine.

The strain was sequenced using the Illumina HiSeq sequencing platform. The paired-end technology of Illumina platform produced a total of 12,884,022 paired-end reads of 100 bp. The next-generation sequencing quality control (NGS QC) toolkit version 2.3 (5) was used to filter high-quality data for the genome assembly. A total of 11,499,584 reads were generated and assembled using the Velvet assembler (version 1.2.10) (6), yielding 216 contigs of 4,791,462 bp and an *N*_50_ value of 90,342 bp. The estimated complete genome size is 4.76 Mb, with a G+C content of 50.65%. Genome annotation was performed using the Rapid Annotations using Subsystems Technology (RAST) server (7), which predicted a total of 4,611 protein-coding sequences, 73 pseudogenes, 72 tRNAs, and 3 rRNA clusters. The NIVEDI-P44 strain belongs to multilocus sequence type (MLST) 746 (ST746). Plasmid Finder and PLACNET (8, 9) detected three plasmid sequences, *viz*., IncN, ColpVC, and p0111 (IncHI1). Of these, p0111 harbored the *tet*(A) gene (encodes tetracycline resistance). PHAST analysis (10) detected 3 intact phages totaling 117.9 kb, which accounts for 2.46% of the total genome.

Analysis by ResFinder version 2.1 (11), ARDB (12), and CARD (13) revealed the presence of the multiple antibiotic resistance genes *bla*_DHA-1_ (class C β-lactamase resistance), *mph*(A) (macrolide resistance), *strA* and *strB* (streptomycin resistance), *dfrA14* (trimethoprim resistance), *sul-2* (sulfonamide resistance), and *qnrS1* (quinolone resistance).

In addition, genes encoding the major facilitator superfamily (MFS), the resistance-nodulation-division (RND) family, multidrug resistance protein A (ErmA), multidrug transporter (MdtABCD), multiple antibiotic resistance protein (Mar ABCR), and the multidrug and toxic compound extrusion (MATE) family of efflux pumps were ascertained. Further, several metal tolerance genes, namely those for nickel, copper, arsenic, cadmium, and zinc, were also identified.

The E. coli strains harboring such genetic resistance determinants and circulating in constantly changing environments contribute to the resistance gene pool, raising the public health threat. Therefore, diligent study of the resistome and mobilome of MDR strains widely disseminated in various environments through comparative genome evaluation will enable us to acquire information about the emergence of resistance.

### Accession number(s).

The draft genome sequences have been deposited in DDBJ/EMBL/GenBank under the accession number LUYD00000000. The version described in this article is the first version.

## References

[1] Cohen ML 2000 Changing patterns of infectious disease. Nature 406:762–767. doi:10.1038/35021206. 10963605

[2] Johnson JR, Russo TA 2005 Molecular epidemiology of extraintestinal pathogenic (uropathogenic) *Escherichia coli*. Int J Med Microbiol 295:383–404. doi:10.1016/j.ijmm.2005.07.005. 16238015

[3] Carattoli A 2008 Animal reservoirs for extended spectrum β-lactamase producers. Clin Microbiol Infect 14:117–123. doi:10.1111/j.1469-0691.2007.01851.x. 18154535

[4] Azarian T, Maraqa NF, Cook RL, Johnson JA, Bailey C, Wheeler S, Nolan D, Rathore MH, Morris JG Jr, Salemi M 2016 Genomic epidemiology of methicillin-resistant *Staphylococcus aureus* in a neonatal intensive care unit. PLoS One 11:e0164397. doi:10.1371/journal.pone.0164397. 27732618 PMC5061378

[5] Patel RK, Jain M 2012 NGS QC toolkit: a toolkit for quality control of next generation sequencing data. PLoS One 7:e30619. doi:10.1371/journal.pone.0030619. 22312429 PMC3270013

[6] Zerbino B, Birney E 2008 Velvet: algorithms for *de novo* short read assembly using de Bruijn graphs. Genome Res 18:821–829. doi:10.1101/gr.074492.107. 18349386 PMC2336801

[7] Aziz RK, Bartels D, Best AA, DeJongh M, Disz T, Edwards RA, Formsma K, Gerdes S, Glass EM, Kubal M, Meyer F, Olsen GJ, Olson R, Osterman AL, Overbeek RA, McNeil LK, Paarmann D, Paczian T, Parrello B, Pusch GD, Reich C, Stevens R, Vassieva O, Vonstein V, Wilke A, Zagnitko O 2008 The RAST server: Rapid Annotations using Subsystems Technology. BMC Genomics 9:75. doi:10.1186/1471-2164-9-75. 18261238 PMC2265698

[8] Carattoli A, Zankari E, García-Fernández A, Voldby-Larsen M, Lund O, Villa L, Møller-Aarestrup F, Hasman H 2014 *In silico* detection and typing of plasmids using PlasmidFinder and plasmid multilocus sequence typing. Antimicrob Agents Chemother 58:3895–3903. doi:10.1128/AAC.02412-14. 24777092 PMC4068535

[9] Lanza VF, de Toro M, Barcia PG, Mora A, Blanco J, Coque TM, Cruz F 2014 Plasmid flux in *Escherichia coli* ST131 sublineages, analyzed by plasmid constellation network (PLACNET), a new method for plasmid reconstruction from whole genome sequences. PLoS Genet 10:e1004766. doi:10.1371/journal.pgen.1004766. 25522143 PMC4270462

[10] Zhou Y, Liang Y, Lynch KH, Dennis JJ, Wishart DS 2011 PHAST: a fast phage search tool. Nucleic Acids Res 39:W347–W352. doi:10.1093/nar/gkr485. 21672955 PMC3125810

[11] Zankari E, Hasman H, Cosentino S, Vestergaard M, Rasmussen S, Lund O, Aarestrup FM, Larsen MV 2012 Identification of acquired antimicrobial resistance genes. J Antimicrob Chemother 67:2640–2644. doi:10.1093/jac/dks261. 22782487 PMC3468078

[12] Liu B, Pop M 2009 ARDB—Antibiotic Resistance Genes Database. Nucleic Acids Res 37:D443–D447. doi:10.1093/nar/gkn656. 18832362 PMC2686595

[13] McArthur AG 2013 The Comprehensive Antibiotic Resistance Database. Antimicrob Agents Chemother 57:3348–3357. doi:10.1128/AAC.00419-13. 23650175 PMC3697360

